# Corporate social responsibility, financial fraud, and firm's value in Indonesia and Malaysia

**DOI:** 10.1016/j.heliyon.2022.e11907

**Published:** 2022-11-25

**Authors:** Tarjo Tarjo, Alexander Anggono, Rita Yuliana, Prasetyono Prasetyono, Muh Syarif, Muhammad Alkirom Wildan, Muhammad Syam Kusufi

**Affiliations:** Faculty of Economics and Business, Universitas Trunojoyo Madura, Indonesia

**Keywords:** Corporate social responsibility, Financial fraud, Firm value, Indonesia, Malaysia

## Abstract

The purpose of this research is to determine if financial fraud can lessen the direct impact of Corporate Social Responsibility (CSR) on firm value. Proxies of the CSR are fourth-generation of the Initiative Global Reporting, religiosity, Philanthropy, Voluntary Environmental Disclosure Index, and ISO 26000. The company's value proxy is Price Book Value and Profit Margin. At the same time, the proxy of financial fraud is the F-Score model. This researcher researches mining companies engaged in Indonesia and Malaysia's oil and gas sectors. This study uses a fixed effect model based on the Hausman diagnostic test statistics. This research reveals evidence that financial fraud can reduce the impact of CSR on a firm's value.

## 1 Introduction

Many researchers have carried out studies on Corporate Social Responsibility (CSR). For example, recent studies on CSR associate CSR with companies conducting IPOs (Arenas-Parra and Álvarez-Otero, 2020), politics and resource bases (Rauf et al., 2020), perception of product market and corporate value (Bardos et al., 2020), disclosure of mandatory non-financial (Jackson et al., 2020), institutional ownership and value (Ur Rehman et al., 2020), government involvement (Xu et al., 2020), increased emissions (Fukada and Ouchida 2020), and financial fraud (Liao et al., 2019). However, research on CSR to explain financial fraud and a firm value is still rarely done.

Previous studies have found that CSR can increase the value of companies (Borghesi et al., 2019; Buchanan et al., 2018; Chang et al., 2019; Gherghina and Vintilă, 2016; Harun et al., 2020; Pham and Tran, 2020; Zolotoy, O'Sullivan and Chen, 2019; NGUYEN, 2021; Saeidi et al., 2021). Slightly different from the view (D'Amato and Falivena 2020), CSR harms the firm's value if applied by companies with small sizes and new ones. Consider CSR as just an actor who covers the spectacle of the company (Chen and Gavious, 2015). Asserts that companies can use CSR to change people's view of fraud behavior (Saxena, 2014). Also, fraud can lower the company's value (Elviani et al., 2020; Sumiyana et al., 2019).

Furthermore, fraud can cause the company's reputation (Dang et al., 2020; Devi et al., 2021; Haß et al., 2019; Hernawati et al., 2021) and the company to suffer losses (Karpoff and Lott, 1993). Internal fraud is the leading actor in the firm's value decline (Eckert et al., 2019; Sakti et al., 2020; Tarjo et al., 2021). In addition to internal factors, the securities fraud scandal can also cause affected companies to lose most of their market value, lowering the Firm's Value. According to such ideas, fraud can reduce the impact of CSR on the value of firm. As a consequence, if there is financial fraud in the organization, the impact of the CSR on the Firm's Value may be diminished.

This study is novel since previous research exploring the impact of CSR on the value of firm with financial fraud as a moderating variable is still scarce. In addition, research that examines the impact of CSR on financial fraud is still based on surveys (primary data), while research using secondary data derived from financial statements is still rarely done. This study chose to use secondary data because the company has officially announced the data, open to the public so that its validity is more reliable (Church, 2002; Liao et al., 2019) than research using surveys (primary data) which has a high potential for bias (Scholderer et al., 2005).

Another difference is that this study uses two countries, namely Indonesia and Malaysia. In addition, this study will use five CSR measurement proxies, i.e., Global Reporting Initiative fourth-generation (GRI-G4), Religiousness, philanthropy, Voluntary Environmental Disclosure Index (VEDI), and ISO 26000, which are still rarely combined in one study. The last difference is that this study adds a moderating variable, namely financial fraud. This argument is motivated by the many previous research debates and the high potential for financial fraud in Indonesia and Malaysia (ACFE, 2018), so the potential for financial fraud will undoubtedly affect the implementation of CSR. So, the potential for financial fraud can provide a differentiator and a solution to the debate in previous research.

This study included 110 samples of annual reports and sustainability reports from Indonesian and Malaysian oil and gas sector businesses. The researcher chose the oil and gas sector because this industry suffered considerable losses from 2019 to 2020 (ACFE 2020). In addition, both countries suffered heavy losses due to fraud (ACFE, 2018). In addition, the two countries also have problems implementing CSR, which is still low, due to the tendency for oil and gas companies to only fulfill obligations (Nasir et al., 2015; Retnaningsih, 2015). Therefore, it is appropriate to research oil and gas companies in Indonesia and Malaysia.

This study employs a quantitative approach, utilizing panel data regression to examine the influence of financial fraud moderation on CSR and corporate value. CSR in this study uses five proxies, namely the Global Reporting Initiative (GRI), and Religiosity, philanthropy (Bond et al., 2014; Febrianny et al., 2020; Zheng and Chen, 2017), Voluntary Environmental Disclosure Index (VEDI), and ISO. 26000 (Chakroun et al., 2019). Then for financial fraud using the Dechow F-Score (Dechow et al., 2011). Meanwhile, the firm's value uses two proxies: price to book value and profit margin (Bardos et al., 2020).

This research adds new literature to the science of accounting and finance. This research shows how CSR relates to corporate values and the influence of moderation of financial fraud on CSR and corporate matters. This research also contributes literature on both countries (Indonesia and Malaysia); by comparing the two countries, we can find something different from the study in general. The differences in characteristics of the two countries will lead to unique and exciting research. This research will provide empirical evidence that CSR can increase the firm's value, financial fraud will cause the firm's value to decrease, and financial fraud can mitigate the impact of CSR on corporate value. Research findings improve CSR reporting and the accuracy of CSR programs, CSR-based fraud reporting, and anti-fraud combined with CSR in Indonesia and Malaysia. In addition, this research also provides research opportunities for researchers in the future.

The composition of the first paper is an introduction, and the second section is a literature review and the formulation of hypotheses. The third section then goes on to the research methodologies, which include data, hypothesis testing, and subsequent robustness testing. At the same time, the fourth section explains the results of hypothesis testing, analysis results, and discussion. The final segment contains the conclusions, limitations, and recommendations for further study.

## 2 Literature review

### 2.1 Stakeholder theory

A stakeholder would be any person or group that exerts influence on one another in order to attain corporate objectives (Freeman, 1984). Furthermore, the stakeholders can be shareholders, employees, customers, or the community (Preston and Sapienza, 1990). Stakeholder theory states that organizational management should be able to account for its stakeholders by carrying out activities deemed necessary by its stakeholders and reporting information. One example of accountability is CSR. Wherewith CSR, companies can take responsibility and also report to stakeholders.

The firm's value may be improving as a result of the company's accountability through CSR. CSR may boost a company's comparative value. The positive effect of CSR is getting higher the importance of companies in companies that have good religious' norms. Found that the small amount of CSR influence on the value of a company depends on the economic condition. In addition, CSR improves companies' reputation and performance (Pham and Tran, 2020).

On the other hand, CSR can distract the public and investors from covering fraud within the company (Saxena, 2014). Fraud will impact investors' decreasing level of investment and the community's welfare (Huda 2019). These two factors lowered the impact of CSR on the firms' value.

### 2.2 Hypothesis

CSR is made up of four parts: economics, law, ethics, and charity (Carrol 1979). This study bases CSR on five proxies: GRI, religiosity, generosity, VEDI, and ISO26000. The two representatives discuss developing societies as proxies for economic components: GRI and ISO26000. VEDI denotes law because it discusses laws or rules regarding environmental preservation (Clarkson et al., 2008, 2013; Plumlee et al., 2015). These components make business operations conducted and can affect the community. Also, I agree with the argument that CSR could improve the community's welfare. The community's increasing interest will enhance the company's reputation (Pham and Tran, 2020). In addition, according to some academics, CSR has a major influence on a company's value (Borghesi et al., 2019; Buchanan et al., 2018; Chang et al., 2019; Gherghina and Vintilă, 2016; Harun et al., 2020; Pham and Tran, 2020; Zolotoy et al., 2019; Saeidi et al., 2021). Thus, the implementation of CSR can increase the company's value and its shareholders. In addition, CSR can also increase accountability and transparency to become a medium for corporate responsibility to stakeholders (Pham and Tran, 2020). But most importantly, the implementation of CSR can increase the value of the company's shares, making every stakeholder happy (Pérez et al., 2020).

CSR can indeed positively impact the firm's value (Borghesi et al., 2019; Buchanan et al., 2018; Chang et al., 2019; Harun et al., 2020; Zolotoy et al., 2019). However, this cannot be generalized. Since some parties are mainly institutional investors, assume CSR is just an actor used to cover the entire corruption of the company (Chen and Gavious 2015). CSR is indeed a positive thing but still not enough to cover the whole crime of the company. I also agreed with the argument because the public argued that CSR is used to divert corporate ugliness. It even confirms that there are still many CSR from oil and gas companies that are not on target and not transparent, so there is potential fraud. Instead of improving the community's welfare but does not have the slightest impact on the community. In addition, research (Elviani et al., 2020; Sumiyana et al., 2019) states that financial fraud can reduce firms' value.

Hence, fraud in a company will lead to a decrease in investment, losses, and a bad reputation in the community. Furthermore, this decrease in the amount of investment and lousy reputation will decrease the positive influence caused by CSR activities on the firm's value. On either side, fraud can erode stakeholder confidence in the organization. CSR should be used as a medium for accountability to stakeholders, but using CSR to commit fraud; causes stakeholders to be disappointed with the company's performance and ultimately hurts company value. As a consequence, the possibility of fraud can reduce the impact of CSR on firm value. The researcher suggests the following hypothesis based on these arguments:

H1 CSR has a significant impact on the firms' value.

H2 Financial fraud weakens the direct impact of CSR on the firms' value.

## 3 Research methods

### 3.1 Sample

This study included 110 samples of annual reports and sustainability reports from Indonesian and Malaysian oil and gas sector businesses from 2010 to 2019. Data sources for yearly reports and sustainability reports include the Indonesia Stock Exchange (https://www.idx.co.id), the Malaysian Stock Exchange (https://www.bursamalaysia.com), and the official websites of each firm.

### 3.2 Variables and the measurements

In this study, the dependent variable is firm value, which is assessed using two proxies, namely PBV and PM. This study uses PM because some companies are not public companies, so calculating the company's value does not involve shares, so the use of PM is appropriate (Bardos et al., 2020). PBV calculation using a formula based is as follows:

(1) $$\mathrm{PBV}=\frac{Market\;Value\;of\;the\;Company}{Company\;Book\;Value}$$

PM calculation using a formula based is as follows:

(2) $$M=\frac{Net\;Profit}{Sales}$$

The independent variable in this study is CSR. CSR proxies in this study include GRI, Religiosity, philanthropy, VEDI, and ISO 26000 (Chakroun et al., 2019).

GRI formula based on the issue of the item issued by GRI is GRI-G4/. GRI-G4 consists of three major themes, namely economic, social and environmental. The formula used is:

(3) $$GRI=\frac{Number\;of\;items\;in\;the\;annual\;report}{91}$$

Religiosity can be proximate to ICSR (Khurshid et al., 2014). The following is the ICSR formula (Othman et al., 2009) in this study. ICSR consists of six themes, i.e., financial and investment, product and service, staffing, social, environmental, and corporate governance. The formula used is:

(4) $$ICSR=\frac{Number\;of\;items\;in\;the\;annual\;statements}{43}$$

The philanthropic formula refers to several studies (Bond et al., 2014; Febrianny et al., 2020; Zheng and Chen, 2017). Philanthropy can come from donations, charity, zakat, social assistance, and community assistance. The formula used is:

(5) Philanthropy=Ln(Philanthropy)

Researchers used the VEDI formula (Clarkson et al., 2008, 2013; Plumlee et al., 2015) in this study. VEDI has seven themes: governance structures and management systems, credibility, environmental performance indicators, ecological expenditures, vision and strategy claims, ecological profiles, and initiatives. The formula used is:

(6) $$VEDI=\frac{Number\;of\;items\;in\;the\;annual\;report}{95}$$

Researchers used the ISO26000 Formula (Chakroun et al., 2019) in this study. The researcher chose this calculation because Indonesia and Malaysia have different ISO26000 regulations, so they chose the latest ISO26000 formula that is internationally applicable. ISO26000 consists of seven themes: Corporate governance, Human Rights, Employment practices, Environment, Fair operating practices, consumer issues, and community engagement and development. The ISO26000 formula used is:

(7) $$ISO\;26000=\frac{Number\;of\;items\;in\;the\;annual\;report}{44}$$

The moderating variable in this study is financial fraud, in which the researcher chose the Dechow F-Score model (Dechow et al., 2011). To calculate the F-Score is as follows:

(8) Fscore=AccrualQuality+FinancialPerformance

(8.1) $$Accrual\;Quality=\frac{WC+NCO+FIN}{Average\;Total\;Asset}$$

(8.2) FinancialPerformance=ΔReceivable+Δinventories+Δcashsales+Δearnings

### 3.3 Empirical model

This study uses panel data regression analysis to test all hypotheses. Researchers chose panel data analysis because this research data combines time series data and cross-section data, so this analysis is the most suitable for testing hypotheses (Ali and Anufriev, 2020; Oscar, 2010). The research panel data regression used three analytical techniques, namely Pooled Ordinary Least Squares (POLS), Fixed Effect Model (FEM), and Random Effects Model (REM).

(9) $$PBV_{it}=\beta_{0}+\beta_{1}GRI_{it}+\beta_{2}Religiosity_{it}+\beta_{3}Philanthropy_{it}+\beta_{4}VEDI_{it}+\beta_{5}ISO26000_{it}+\alpha_{it}+u_{it\;}$$

(10) $$PBV_{it}=\beta_{0}+\beta_{1}\;GRI_{it}+\beta_{2}\;Religiosity_{it}+\beta_{3}\;Philanthropy_{it}+\beta_{4}\;VEDI_{it}+\beta_{5}\;ISO26000_{it}+\beta_{6}\;GRI*FRAUD_{it}+\beta_{7}\;Religiosity*FRAUD_{it}+\beta_{8}\;Philanthropy*FRAUD_{it}+\beta 9VEDI*FRAUD_{it}+\beta_{10}\;ISO26000*FRAUD_{it}+\alpha_{it}+u_{it}$$

(11) $$PM_{it}=\beta_{0}+\beta_{1}GRI_{it}+\beta_{2}Religiosity_{it}+\beta_{3}Philanthropy_{it}+\beta_{4}VEDI_{it}+\beta_{5}ISO26000_{it}+\alpha_{it}+u_{it}$$

(12) $$PM_{it}=\beta_{0}+\beta_{1}\;GRI_{it}+\beta_{2}\;Religiosity_{it}+\beta_{3}\;Philanthropy_{it}+\beta_{4}\;VEDI_{it}+\beta_{5}\;ISO26000_{it}+\beta_{6}\;GRI*FRAUD_{it}+\beta_{7}\;Religiosity_{it}*FRAUD_{it}+\beta_{8}\;Philanthropy*FRAUD_{it}+\beta 9VEDI*FRAUD_{it}+\beta_{10}\;ISO26000*FRAUD_{it}+\alpha_{it}+u_{it}$$

Where: PBV is the price to book value, PM is the profit margin, GRI is an index of GRI-G4, Religiosity is an index of Islamic Corporate Social Responsibility (ICSR), Philanthropy is the natural logarithm of a donation or charity or social fund, VEDI is an index of VEDI, ISO26000 is an index of ISO260000, FRAUD is the value of the Dechow F-Score model, β_0_ is constant, β1- β_10_ a regression coefficient. *u*_it_ (i = 1,…n) denotes unknown intercepts for each entity (n entity-specific intercepts), and *u*_it_ denotes error terms.

Fixed Effect Model to evaluate the net influence of predictors on outcome variables, FEM removes the effect of time-invariant features (Ali and Anufriev, 2020; Oscar, 2010). FEM also believes that the time-invariant property is unique because it does not correlate one variable with another variable property (Ali and Anufriev, 2020; Oscar, 2010). If the researcher performs correlation errors, the FEM becomes unsuitable because it can make wrong conclusions; thus, it is necessary to model such a relationship with REM. This method forms the basis for the Hausman test (Ali and Anufriev, 2020; Oscar, 2010). Here are the FEM models:

## 4 Research findings

This study's conclusions include descriptive statistics, correlation tests, classical assumptions, endogeneity test, the Hausman test, and hypothesis testing with panel data regression. The following sections present the study's findings in chronological order.

### 4.1 Descriptive statistics

Table 1 will explain the results of descriptive statistical tests.

**Table 1:** Descriptive statistics.

Variable	Sample	Minimum	maximum	Mean	Sd
GRI	110	0.2197	0.6263	0.3997	0.1073
Religiosity	110	0.1860	0.7209	0.5198	0.1119
Philanthropy	110	25.1866	29.7089	26.3517	0.8157
VEDI	110	0.0526	0.5789	0.3275	0.1202
ISO26000	110	0.2727	0.9545	0.6824	0.1389
PBV	110	-2.6478	6,2622	0.7176	1.1666
PM	110	-2.6478	1,9606	0.1732	0.3949
Fraud	110	-14.7801	8.9427	1.0856	2.3533

### 4.2 Correlation test

The goal of this correlation test is to investigate the relationship between each variable. In Table 2, the test results show that CSR correlates with firm value as proxied by PBV. But on the other hand, some CSR proxies are not associated with PM. In addition, the fraud variable has a negative correlation with firm value. Here are the results of the correlation test:

**Table 2:** Correlation test.

	GRI-G4	Religiosity	Philanthropy	VEDI	ISO26000	Fraud
GRI-G4						
Religiosity	0.650∗∗					
Philanthropy	0.059	0.104				
VEDI	0.625∗∗	0.681∗∗	-0.067			
ISO26000	0.738∗∗	0.880∗∗	0.073.	0.620∗∗		
Fraud	0.160	0.347∗∗	-0.035	0.173	0.338∗∗	
PBV	-0.094	-0.309∗∗	0.091	-0.278∗∗	-0.360∗∗	-0.231∗∗
PM	0.126	0.174	-0.010	0.083	0.108	0.127

### 4.3 Classical assumptions test results

This study uses an assumption test to determine whether the research data is feasible to test the hypothesis (Ghozali, 2016). The classical assumption consists of four tests: normality, heteroscedasticity, multicollinearity, and autocorrelation. Furthermore, Table 3 shows the results of classical assumption testing.

**Table 3:** Fixed effect model result.

Variable	VIF	PBV		PM		VIF	PBV		PM	
		Coefficient	t	Coefficient	t		Coefficient	t	Coefficient	t
GRI	2.502	0.759	6.430∗ (0.000)	0.435	4.822∗ (0.000)	1.851	2.003	6.044∗ (0.000)	2.223	4.722∗ (0.000)
Religiosity	5.346	1.178	5.391∗ (0.000)	5.775	2.101∗∗ (0.038)	2.036	1.521	5.541∗ (0.000)	2.766	2.613∗∗ (0.010)
Philanthropy	1.056	0.027	6.487∗ (0.000)	1.467	3.627∗ (0.000)	1.004	0.032	7.091∗ (0.000)	5.225	2.920∗ (0.004)
VEDI	2.209	1.614	4.727∗ (0.000)	2.452	2.037∗∗ (0.014)	1.149	2.130	5.157∗ (0.000)	2.345	2.024∗∗ (0.045)
ISO26000	5.737	0.887	5.318∗ (0.000)	6.315	5.277∗ (0.000)	1.939	0.836	5.027∗ (0.000)	0.405	4.584∗ (0.000)
GRI∗Fraud						1.289	-0.316	-2.088∗∗ (0.039)	-1.205	-3.369∗ (0.001)
Religiosity∗Fraud						1.432	-.0251	-2.016∗∗ (0.046)	-1.223	-2.005∗∗ (0.047)
Philanthropy∗Fraud						1.027	-0.004	-2.523∗∗ (0.013)	-0.147	-2.015∗∗ (0.046)
VEDI∗Fraud						1.173	-0.387	-2.146∗∗ (0.034)	-0.199	-2.227∗∗ (0.028)
ISO26000∗Fraud						1.388	-0.131	-1.991∗∗ (0.049)	-0.105	-3.045∗ (0.002)
Prob. (Jarque-Bera)		0.110		0.065			0.053		0.709	
Breusch-Pagan-Godfrey		0.107		0.158			0.114		0.216	
Durbin-watson stat		0.948		0.513			0.951		0.659	
Durbin-Wu-Hausman		0.660		0.685			0.066		0.426	
Hausman Test Decision		0.000∗ Accepted		0.000∗ Accepted			0.000∗ Accepted		0.000∗ Accepted	

Note: p-value are in round brackets (); ∗ and ∗∗ connotes 1% and 5% Level of significance.

PBV: Price Book Value; and PM: Profit Margin.

The Jarque-Bera probability value indicates the normality test. If the probability value is >0.05, the conclusion is that the residual data has a normal distribution. Table 3 shows that all variables and research models have probability values >0.05. These results prove that the residual data has a normal distribution.

Furthermore, multicollinearity test using Variance Inflation Factor (VIF). If the VIF value >1, the data is free from multicollinearity. The test results in Table 3 show that the research model is free from multicollinearity.

The heteroscedasticity test uses the Breusch-Pagan-Godfrey probability value. If the Breusch-Pagan-Godfrey value >0.05, the data is classified as non-heteroscedasticity. The test results show that the Breusch-Pagan-Godfrey value is >0.05 (Table 3). These results prove that the data used is non-heteroscedasticity data.

Finally, the autocorrelation test uses the Durbin-Watson value. Based on the test results in Table 3, the Durbin-Watson value is between DW (du) and DW (dl). These findings demonstrate that there is no autocorrelation in the data. As a consequence, the results of the classical assumption tests demonstrate that the data matched the conditions for doing panel data regression testing.

### 4.4 Endogeneity result

The use of the endogeneity test is to ensure that the research model is free from the influence of endogeneity or not (Ejemeyovwi et al., 2019; Oloyede et al., 2021). Durbin-Wu-Hausman test (Table 3) to test for endogeneity (Ejemeyovwi et al., 2019; Oloyede et al., 2021). Based on the results of the endogeneity test, all Durbin-Wu-Hausman values were greater than 5%. Thus, this research model is free from the influence of endogeneity.

### 4.5 Hausman test

This section presents the results of the Hausman test. A rule of thumb for deciding which estimate to interpret between FEM and REM by the Hausman test (Ejemeyovwi et al., 2019; Oloyede et al., 2021). Researchers analyzed the effect of CSR (GRI, religiosity, philanthropy, VEDI, and ISO26000) on firm value (PBV and PM) by moderating financial fraud.

Table 3 presents all FEM tests. FEM test results show that all models are fixed-effect models because the hausman test show a random cross-section probability value below 5%. The Hausman test shows that this research model is a fixed-effect model.

### 4.6 Hypotheses test results

Table 3 presents the results of testing the effect of CSR on firm value (PBV and PM) and the moderating effect of financial fraud on CSR with firm value. The test results in Table 3 show that GRI, religiosity, philanthropy, VEDI, and ISO26000 significantly positively affect PBV and PM. The test results in Table 3 show that GRI, religiosity, philanthropy, VEDI, and ISO26000 significantly positively affect PBV and PM. This finding proves that the significance value of all CSR proxies is 0.000 to PBV. On the other hand, the coefficients and t-statistics of each proxy have positive values. Based on these tests, the researchers concluded that CSR significantly positively affects firm value. Thus, the test results support the first hypothesis.

In Table 3, presents the results of testing the effect of CSR on firm value moderated by financial fraud. Based on the test results in Table 3, the CSR variables proxied by GRI, religiosity, philanthropy, VEDI, and ISO26000 moderated by financial fraud have p-values of 0.039, 0.046, 0.013, 0.034, and 0.049 for PBV. As for the firm's value proxied by PM, each proxy has a significance value of 0.001, 0.047, 0.046, 0.028, and 0.002. In addition, the coefficients and t-statistics of these variables have negative values, meaning that financial fraud can weaken the influence of CSR on firm value. Thus, the test results support the second hypothesis.

### 4.7 Discussion

#### 4.7.1 CSR and Firm's value

This study found that CSR has a positive impact on firm value. CSR proxies which include GRI, religiosity, philanthropy, VEDI, and ISO26000, have a significant p-value and have a positive impact on firm value. This finding supports previous studies which found that CSR can increase firm value (Borghesi et al., 2019; Buchanan et al., 2018; Chang et al., 2019; Gherghina and Vintilă, 2016; Harun et al., 2020; Pham and Tran, 2020; Zolotoy et al., 2019; Saeidi et al., 2021).

It indicates that CSR can positively impact every stakeholder, including investors and the public so that CSR can increase the company's value. In addition, the research findings prove that the implementation of CSR in Indonesia and Malaysia is quite good (Table 1). Because the value of the company's shares rises as a result of excellent CSR implementation, it can improve the company's value and deliver benefits to stakeholders.

According to the findings of the research and discussions, all CSR research factors have a considerable impact on firm value. In general, companies that implement CSR can increase the company's value. Although CSR projected by various proxies should get the same results, CSR has a substantial and beneficial impact on corporate value.

#### 4.7.2 CSR, Firm's value, and financial fraud

This research finds that the company's financial fraud reduces the impact of CSR on the firm's value. Table 3 shows that each CSR proxy moderated by financial fraud has a significant p-value and a negative direction. Thus, the presence of financial fraud weakens the influence of CSR on firm value.

This study found empirical evidence that investors began to revoke investments in the company with the financial fraud. Then, prospective investors started to be disinterested in investing in companies with indications of financial fraud. This influence led to a decrease in the firms' value (Elviani et al., 2020; Sumiyana et al., 2019). In addition, financial fraud can also cause a decline in the company's reputation, can even damage it, and make the company lose money (Karpoff and Lott, 1993). So, with the financial fraud in the company making the positive value of CSR decrease, there may even be indications that financial fraud in CSR can even decrease the firm's value.

From the community's perspective, CSR policy does not lead directly to the community's welfare to not improves the community's interest. The most important thing is the issue of transparency of CSR funds. This lack of clarity will cause much fraud in using these funds (Huda 2019). Subsequently, it will decrease the positive value of CSR to the reputation and weight of the company. Of course, this happens because the community does not provide a positive impact and the potential for misappropriation of CSR funds is less transparent.

Therefore, financial fraud harms the company and its stakeholders. We have proven that financial fraud can negatively impact companies because financial fraud can reduce transparency and imprecise CSR implementation, reducing company value. Financial fraud also makes stakeholders feel betrayed so that stakeholders are not happy with what has happened. As a result, the likelihood of fraud may reduce the impact of CSR adoption on firm value.

## 5 Conclusion, suggestions, and implication

The study analyzed data from 110 Indonesian and Malaysian oil and gas energy companies because both countries have a high potential for fraud and ineffective CSR implementation.

The first finding of this study is that CSR can increase the firms' value. The integration of CSR components creates prosperity for all stakeholders. Implementing CSR impacts increasing accountability, transparency, and company value so that the value of company shares increases.

The main finding of this study is that financial fraud can weaken the influence of CSR on firm value. Financial fraud in the company harmed the stakeholders, so the company's value decreased. So, this study demonstrated that financial fraud might reduce the influence of CSR on firm value.

The limitations of this study lie in the lack of samples used and focus only on oil and gas energy companies. If seen in 2020, Oil and gas energy firms are only a subset of the mining sector. In addition, firm value uses only two proxies, and financial fraud uses only one representative. Finally, this study did not use control variables.

The advice for further research is to reproduce the research sample and use all sub-sectors in the mining sector. The addition of a proxy to the enterprise value is like the use of Tobin's Q. In addition, future research can add proxies for financial fraud, for example, using the Beneish M-Score model. In addition, this study strongly suggests mixed-method research by using secondary data from the company's annual financial statement and primary data acquired from the community around the affected companies and conducting interviews with the surrounding community. The research will be more accurate because it results from two perspectives: the company and the surrounding community. Hence, it can provide input to companies and central and local governments and villages. Finally, we suggest adding control variables such as size, financial ratios, and non-financial ratios such as company age.

The results of this study provide practical implications in the form of improving the transparency of CSR reporting, improving regulations regarding CSR, and anti-fraud policies. In addition, this study signals to stakeholders and the government about the potential dangers of fraud in CSR practices. This study also provides theoretical implications regarding CSR research and financial fraud. Thus, this study provides research opportunities for future researchers and can be additional literature on CSR.

## Declarations

### Author contribution statement

Tarjo Tarjo; Alexander Anggono: Conceived and designed the experiments; Performed the experiments; Contributed, reagents, materials, analysis tools or data; Analyzed and interpreted the data; Wrote the paper.

Prasetyono Prasetyono; Rita Yuliana; Muhammad Alkirom Wildan; Muh Syarif; Muhammad Syam Kusufi: Conceived and designed the experiments; Contributed reagents, analysis tools or data; Analyzed and interpreted the data; Wrote the paper.

### Funding statement

Dr. Tarjo Tarjo was supported by Universitas Trunojoyo Madura, Number 159/UN46.4.1/PT.01.03/2020.

### Data availability statement

The authors do not have permission to share data.

### Declaration of interest's statement

The authors declare no conflict of interest.

### Additional information

No additional information is available for this paper.
